# The effects of photoperiod and temperature-related factors on maize leaf number and leaf positional distribution in the field

**DOI:** 10.3389/fpls.2023.1006245

**Published:** 2023-02-10

**Authors:** Honggen Xu, Bo Ming, Keru Wang, Jun Xue, Peng Hou, Shaokun Li, Ruizhi Xie

**Affiliations:** Key Laboratory of Crop Physiology and Ecology, Institute of Crop Sciences, Chinese Academy of Agricultural Sciences, Ministry of Agriculture and Rural Affairs, Beijing, China

**Keywords:** photoperiod, temperature-related factors, maize, leaf number, leaf positional distribution

## Abstract

Quantifying the effects of various environmental conditions on maize leaf number is essential to understanding the environmental adaptations and population structure of maize plants and for enhancing maize productivity. In this study, seeds of three temperate-adapted maize cultivars, each belonging to different maturity classes, were sown on eight different dates. Sowing dates ranged from the middle of April to early July, which allowed us to cover a wide range of environmental conditions. Random forest regression and multiple regression models with variance partitioning analyses were used to assess the effects of environmental factors on the number of leaves and their distributions on maize primary stems. We demonstrated that the total leaf number (TLN) increased in the three cultivars in the following order: FK139 < JNK728 < ZD958, and variations in TLN for each cultivar were 1.5, 1.76, and 2.75 leaves, respectively. The variation in TLN was ascribed to changes in LB (leaf number below the primary ear), which were higher than variations in LA (leaf number above the primary ear). Variations in TLN and LB were mainly affected by the photoperiod during growth stages V7 to V11, and differences in TLN and LB in response to different photoperiods ranged from 1.34 to 2.95 leaves h^-l^. Variations in LA was mainly affected by temperature-related factors. Therefore, the results of this study enhanced our current understanding of key environmental conditions that affect maize leaf numbers, and provides scientific support for the benefits of adjusting sowing dates and selecting suitable cultivars to mitigate the effects of climate change on maize production.

## Introduction

Crop growth is strongly affected by external environmental factors (Craufurd and Wheeler, 2009; Poorter et al., 2016), such as temperature (Lizaso et al., 2018), solar radiation (Shi et al., 2022), water availability (Nakayama and Bucks, 1991) and photoperiod (Craufurd and Wheeler, 2009), all of which ultimately lead to uncertainties for crop production (Tsimba et al., 2013a). Therefore, a great challenge in crop production is identifying critical meteorological factors affecting crop growth and yield. For instance, when the same variety of maize was planted at different latitudes, the resulting TLN varied by 5 or more leaves (Liu et al., 2020). Changes in leaf number are important as they affect key growth and developmental processes, leaf area, and the overall canopy morphology of maize plants, which affects plant biomass accumulation and yield; as such, leaf number can be used to assess regional adaptations among different maize cultivars (Allen et al., 1973; Ellis et al., 1992; Bonhomme et al., 1994; Lafitte and Edmeades, 1997; Li et al., 2016; Liu et al., 2020). Leaf number is also a useful metric for modeling maize phenology and ontogeny (Bonhomme et al., 1991; Birch et al., 1998b).

Maize leaves develop from stem apical meristems, which usually occurs before tassel initiation (Bonhomme et al., 1991). Environmental factors affect the leaf initiation rate and duration, two important traits that ultimately determine the final leaf number (Tollenaar and Hunter, 1983). Many researchers have investigated variations in leaf number between different maize cultivars in response to environmental factors, such as photoperiod and air temperature (Russell and Stuber, 1983; Tollenaar and Hunter, 1983; Warrington and Kanemasu, 1983; Ellis et al., 1992; Liu et al., 2020), diurnal temperature range (Bonhomme et al., 1991), rate of changing daylength (Bonhomme et al., 1991), and incident photosynthetic photon flux density (Tollenaar, 1999).

Most studies investigating changes in maize leaf number in response to environmental factors have been focused on field experiments in multiple locations or altering sowing dates (Bonhomme et al., 1991; Birch et al., 1998a; Giauffret et al., 2000; Liu et al., 2020), however some studies have also used controlled-environment cabinets to more tightly dissect the effects of different environmental factors on maize leaf number (Allison and Daynard, 1979; Chen et al., 2015; Francis et al., 1969; Stevenson and Goodman, 1972; Tollenaar, 1999), and a few others used combined field based and climate controlled experiments (Russell and Stuber, 1985; Mungoma and Pollak, 1991). However, between field based and controlled-environment experiments, controlled-environment studies consistently obtain lower photothermal ratios (the ratio between the daily amount of light and daily temperature), and the correlation between controlled-environment and field phenotypic data is very low (Poorter et al., 2016). Hence, it is questionable whether responses quantified in a controlled environment can accurately reflect field performance (White et al., 2012; Poorter et al., 2016). Furthermore, there are experimental limitations to controlling so many field-related factors; nearly all research conducted under field conditions has focused on just one or two environmental factors for which pertinent information is lacking, and ignored how interactions between multiple meteorological factors can affect maize leaf number.

Many studies have suggested that effective measures to cope with the impending effects of global climate change on maize crop production are to adjust the sowing date to allow for acclimation to increasing temperatures (Huang et al., 2020; Liu et al., 2013b; Zhang et al., 2020). However, altering the sowing date also changes the photoperiod to which maize seedlings are exposed during the growing period, and the effects of this on maize growth and yield have not been accurately studied (Tsimba et al., 2013b). In this study, we conducted a series of sowing date experiments that cover a wide range of temperature, photoperiod, and radiative conditions to explore the comprehensive effects of multiple meteorological factors on variations in maize leaf number. This study’s objectives are to investigate variations in leaf number below the primary ear (LB), leaf number above the primary ear (LA), total leaf number (TLN), and whether differences in LB and LA contribute to TLN. We also aimed to elucidate the effects of multiple meteorological factors on LB and LA in association with TLN, and identify which meteorological factors occurring at which growth stages are vital in determining maize leaf numbers.

## Materials and methods

### Study site, climate, and soil data

Field experiments were conducted from 2018 to 2020 at Xinxiang Experimental Station, Chinese Academy of Agriculture Sciences (35°18′N, 113°54′E), Henan province. Maize was grown almost entirely under irrigated conditions. The average annual number of frost-free days was about 260 d, the average annual accumulated temperature was about 2700-3000 °. The soil texture at the site was clay loam (ISSS Classification, International Soil Science Society) with 12.6 g kg^-1^ of organic material, 61.2 mg kg^-1^ of available N, 16.2 mg kg^-1^ of available P, 110.0 mg kg^-1^ of available K, and a pH of 8.21.

### Experimental design and crop management

The election of the genotypes of this study was mainly based on maize maturity, as this a key trait affecting the distribution of different cultivars within existing production areas, which could be associated with the impact of environmental factors on maize leaf numbers. Therefore, three temperate-adapted maize cultivars, FK139, JNK728, and ZD958, with different maturity periods and germplasm origins (**Table 1**) were planted from 2018 to 2020. The required quantities of ≥0 °-accumulated-temperature through a season of growth for the three hybrids were 2700 °, 2800 °, and 3100 °, respectively.

**Table 1:** The sources of maize cultivars selected for study from 2018–2020.

Cultivar	Parents	Parent name	Pedigree	Heterosis groups	Type
FK139	Male parent	K454	Zha 917×K 161	Lancaster	Temperate
	Female parent	FK334	PH6WC× PH4CV	Reid	Temperate
JNK728	Male parent	Jing 2416	Jing 24 ×5237	Tangsipingtou	Temperate
	Female parent	Jing MC01	X1132x	X group	Temperate
ZD958	Male parent	Chang 7-2	Huangzaosi×V59×Swan1	Tangsipingtou	Temperate
	Female parent	Zheng 58	Ye 478	Reid	Temperate

Treatments for field experiments in each year consisted of eight sowing dates (**Table 2**) to cover a wide range of weather regimes associated with each date and still allow crops time to reach physiological maturity. The eight sowing dates are denoted as: SD1, SD2, SD3, SD4, SD5, SD6, SD7, and SD8.

**Table 2:** The sowing dates for each treatment established each year from 2018 to 2020.

Treatment	2018		2019		2020	
	Sowing date	Days of year	Sowing date	Days of year	Sowing date	Days of year
SD1	19-Apr	109	24-Apr	114	20-Apr	111
SD2	30-Apr	120	4-May	124	30-Apr	121
SD3	10-May	130	14-May	134	13-May	134
SD4	24-May	144	24-May	144	23-May	144
SD5	3-Jun	154	3-Jun	154	2-Jun	154
SD6	13-Jun	164	13-Jun	164	14-Jun	166
SD7	23-Jun	174	23-Jun	174	24-Jun	176
SD8	3-Jul	184	3-Jul	184	4-Jul	186

Two or three maize seeds per hole were planted at a soil depth of 5.0 cm, and the resulting seedlings were thinned at the V3 stage to a target density of 75,000 plants ha^-1^ with an equal spacing of 0.6 m. Each plot was 9 m long and 7.2 m wide, and each consisted of 12 rows. We established a randomized complete block design with three replications for each treatment in this experiment. Before sowing, the plot was finely prepared and moistened. Experimental plots were irrigated after sowing and fertilized according to soil analysis recommendations, typically at rates of 225 kg of N ha^-1^, 173 kg of P_2_O_5_ ha^-1^, and 150 kg of K_2_O ha^-1^. Fertilizers were applied once before sowing to avoid nutrient limitations. Irrigation was applied as required to maintain soil moisture near field capacity to avoid any degree of water stress. Crops were protected from pests, pathogens, and weeds according to local farm management practices.

### Measurements and estimations

#### Leaf stages

Prior to assessing leaf stages and recording maize development, the timing of seedling emergence was recorded from seeds planted in three of the 12 rows (per plot) and within a 1 m length of each row. Once 50% of the viable seeds emerged from the soil, the seedling emergence stage (V1) was recorded. Twenty healthy maize plants were chosen from among the emerged seedlings to assess leaf stages. Leaf stages were defined by the number of leaves with visible tips in a whorl, and the number of visible leaf tips was recorded daily for each treatment (Zadoks et al., 1974). When the n^th^ number of leaf tips in a whorl was observed, a new leaf stage of maize was recorded as Vn (i.e. at stage V1 one leaf tip is visible, and so on). Leaves 6 and 12 were paint-marked to use as references for leaf number.

#### Leaf numbers

Before the first leaf had senesced 20, 50, and 50 plants from the center rows were selected for leaf number counting in the 2018, 2019, and 2020 crop seasons, respectively. Per treatment, plants that appeared uniform in growth at the silking stage were chosen at random and then tagged by red paint for convenient and accurate counting of TLN, LB, and LA.

#### Weather data

Half-hourly data collections were made for the meteorological variables, including air temperature and solar radiation at the field site, using a “WatchDog” Data Logger (Spectrum Technologies, Inc., USA) during the vegetative growth stage of maize. Sensors to measure solar radiation and air temperature were placed 2 m above ground level. Photoperiod (sunrise to sunset and civil twilight) was computed using the method described by Forsythe et al. (1995).

### Data processing and statistical analysis

The number of leaves appearing along the main stem of a plant can be used to examine maize phenology (Zadoks et al., 1974). Here, three counts of leaf numbers (TLN, LB, and LA) were counted prior to maize plants initiating tassel formation (Ellis et al., 1992). Tassel initiation reportedly occurs at a leaf stage that is numerically equal to 50% of the final leaf number per plant (Tollenaar and Hunter, 1983). The average TLNs of our three cultivars, FK139, JNK728, and ZD958, were 17.26, 18.19, and 19.75 leaves, respectively. The final leaf numbers at the tassel initiation stage for each cultivar were approximately 8.6, 9.1, and 9.9 leaves, respectively. The topmost axillary meristem is initiated shortly after tassel initiation (Lejeune and Bernier, 1996). So in this study we focused on the stages from seedling emergence to V11. We divided the different maize developmental stages from seedling emergence (V1) to V11. In order to determine the most accurate sensitivity period, we divided the cycles from V1 to V11 into different developmental stage combinations, such as stages V1 to V2, V1 to V3, V1 to V4, V1 to V5…V2 to V3 stage, V2 to V4 stage, V2 to V5 stage … V10 to V11 stage, and so on. In total, 55 different developmental stage combinations were used to determine the sensitivity of each stage to different weather variables. Combinations of each of the 55 stages and each of the six meteorological factors—mean air temperature (MT), average maximum air temperature (Tmax), average minimum air temperature (Tmin), average temperature range (Tr), photoperiod (PP), and photosynthetically active radiation (PAR)—produced 330 combinations of variables for this study. The effects of weather variables on different developmental stages correlated strongly with one another, which could lead to a serious multicollinearity problem in the traditional statistical models, for instance stepwise multiple regression and linear regression analyses haven’t eliminated latent multicollinearity. Random forest is not influenced by multicollinearity issues (Dormann et al., 2013). Hence, in this study to avoid multicollinearity issues in the data analysis, the random forest regression model was used to determine the combined factors that were likely responsible for variation in our three counts of leaf numbers (Dormann et al., 2013; Trivedi et al., 2016). For the random forest models, we used each of the three counts as response variables and the 330 combined variables as predictors. We used percentage increases in the MSE (mean squared error) of variables to assess the importance of the combined factors. Higher MSE% values indicated greater involvement of a variable (Breiman, 2001). The 10 most critically important combined factors were selected based on the MSE%.

Significant effects of each predictor on response variables were estimated using the R package ‘rfPermute’ (version 4.1.1; http://www.r-project.org/).

A multiple regression model with variance partitioning analysis was also used to evaluate the importance of the top 10 critically important combined factors identified by the random forest analysis, and to identify key combined factors influencing variations observed in any of the three leaf counts using the lm and calc.relimp functions in the “relaimpo” R package (version 4.1.1; http://www.r-project.org/).

Analyses of variance (ANOVA) was used to estimate the effects of the sowing date, year, cultivar, and their correlations with the three maize leaf numbers of interest using R (version 4.1.1; http://www.r-project.org/). Significant effects were determined by *P*-values from the F-test and variation partitioning was determined based on the sum of squares. The packages “ggplot2” (version 4.1.1; http://www.r-project.org/), Microsoft Visio 2003, and Excel 2016 were used to produce figures. In order to present the data concisely, the notations *, **, and *** are used to indicate significance levels at 0.01 < *P* < 0.05, 0.001 < *P* < 0.01, and *P* < 0.001, respectively. In addition, ns refers to *P* ≥ 0.05.

## Results

### Meteorological conditions and crop development

**Figure 1:**
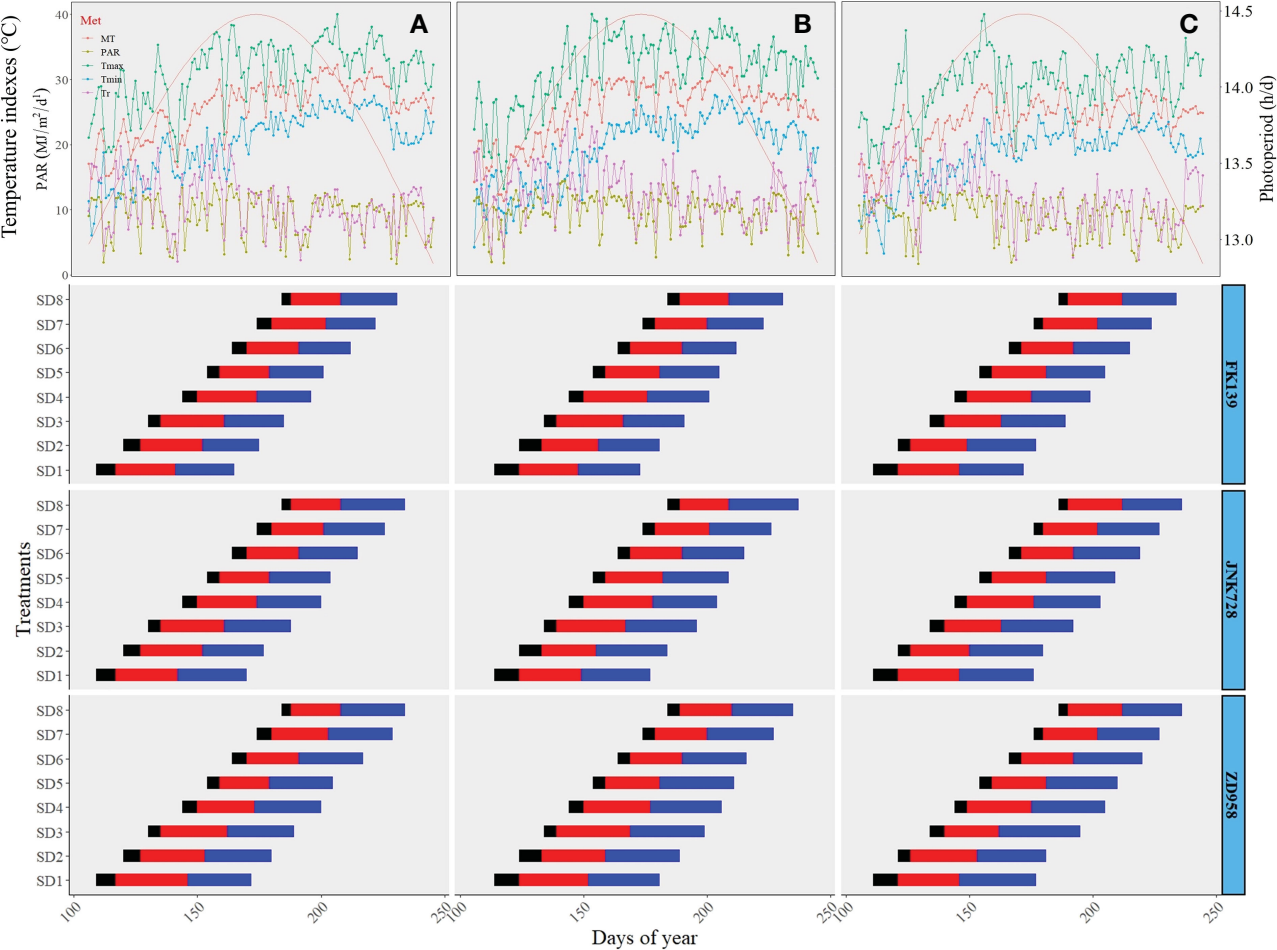
Time-course of meteorological conditions during maize vegetative growth. (**A–C**) respectively represent the meteorological conditions of 2018, 2019, and 2020, and the below horizontal bars indicate the duration (days) of maize phenological periods: black-bars indicate the time from sowing to emergence (V1), red-bars indicate the time fromV1 to V11 (The period of V1 to V11 nearly the time of maize leaf differentiation), blue-bars indicate the time fromV11 to silking (Xu et al., 2023).

Meteorological conditions measured during crop vegetative growth in the 2018 to 2020 maize growing seasons are shown in **Figure 1**. The time from emergence to the silking period was between 42-60 d for all experiments, and the duration of this timeframe became significantly shortened with later sowing dates. Maize plants developed from the middle of April to early July, and were covered to a broad range of photoperiod, thermal, and radiative conditions. More specifically, during the studied developmental period from seedling emergence to tassel initiation (stages V1 to V11, which occurred at about 20 days after seedling emergence), the average PP ranged from 13.76 to 14.46 h/d, the MT ranged from 21.20 to 29.38 °, the Tmax ranged from 26.93 to 34.47 °, the Tmin ranged from 10.02 to 26.70 °, the Tr ranged from 8.96 to 16.21 °, and the PAR ranged from 8.20 to 11.18 MJ m^-2^d^-1^ (**Figure 1**). Meteorological conditions exhibited similar values and trends during the period from seedling emergence to silking.

### Variation of leaf numbers

**Figure 2:**
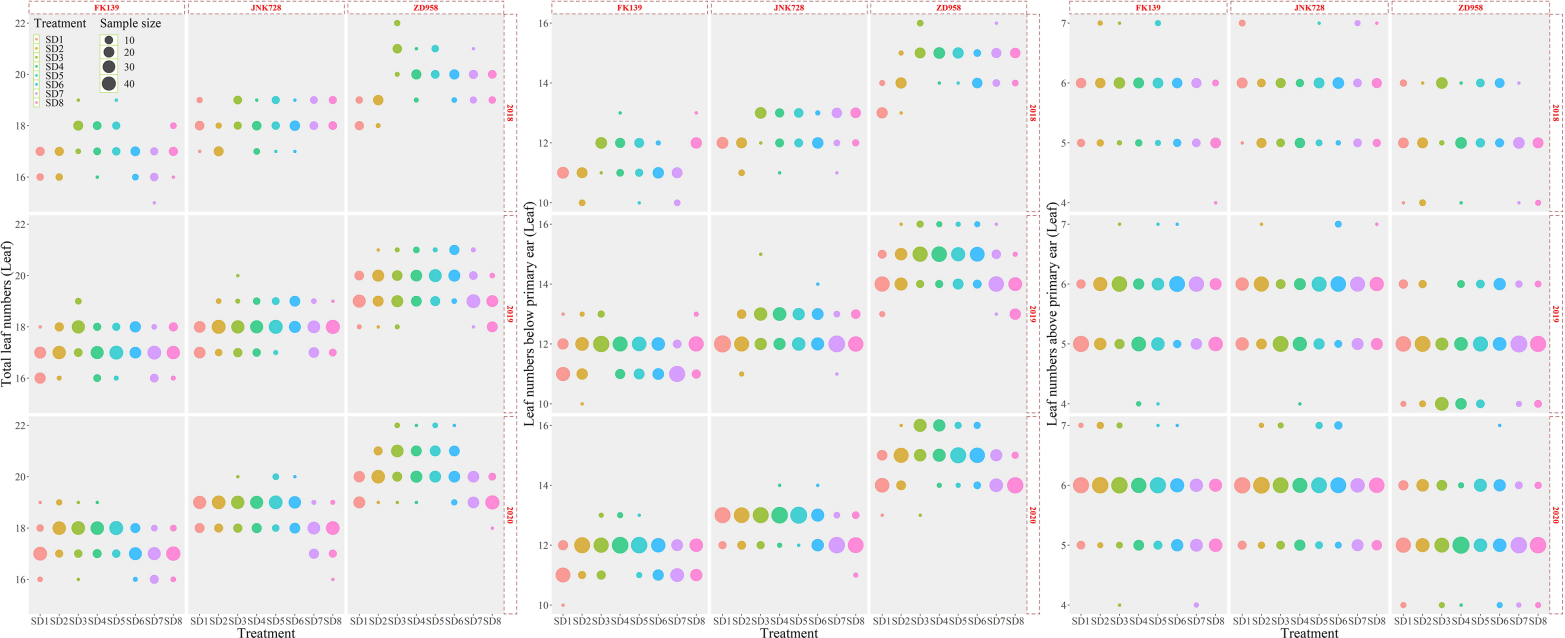
Variation observed in leaf counts relative to sowing dates. A, variation in TLN; B, variation in LB; C, variation in LA.

**Figure 3:**
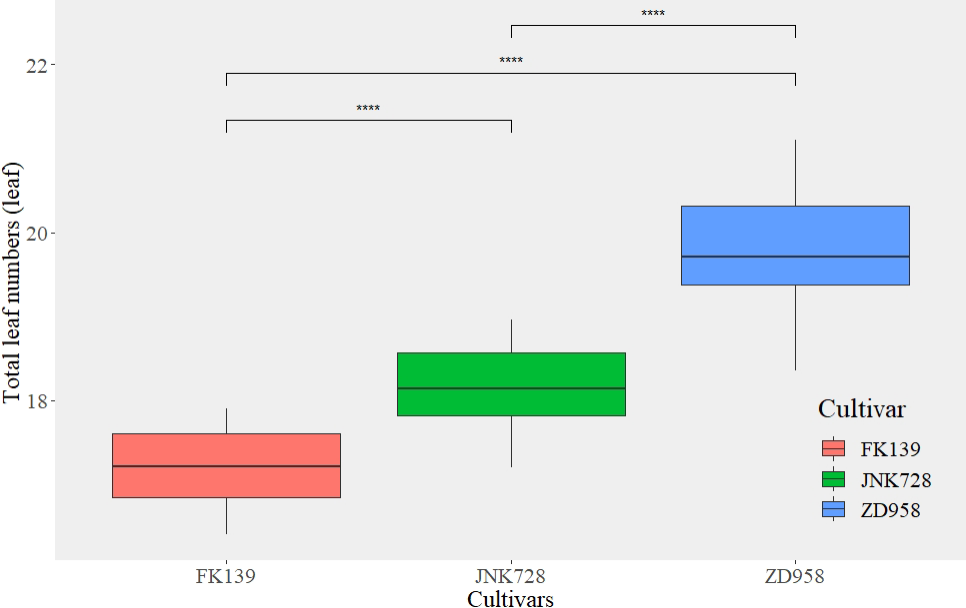
The total leaf number among different maize cultivars. ****are used to indicate significance levels at P < 0.0001.

As shown in **Figure 2**, the TLN ranged from 16.40 to 21.10; the mean value of the TLN was 18.39 across all cultivars and treatments (n=2880). The TLN significantly differed among cultivars (**Figure 3**); the TLNs of FK139, JNK728, and ZD958 were 17.27 (n = 960), 18.19 (n = 960), and 19.76 (n = 960) leaves, respectively. LB exhibited the same trends in variation observed for TLN, wherein it started out high and decreased in accordance with the delay in sowing date. The LB ranged from 10.80 to 15.48 leaves, and the mean value of LB was 12.86 (n = 2880) leaves for all treatments. The LB for each cultivar significantly increased in the following order: 11.57 (FK139, n = 960), 12.41 (JNK728, n = 960), and 14.61 (ZD958, n = 960). The TLN and LB of ZD958 were significantly higher than the corresponding values of the other two cultivars. Variations in LA were lower than the observed variations in TLN and LB. The average LA was 5.51 leaves (n = 2880) among all treatments. The mean LA for each cultivar significantly increased in the following order: 5.14 (ZD958, n = 960), 5.66 (FK139, n = 960), and 5.71 (JNK728, n = 960). In contrast to the TLN and LB data where FK139 had the lowest mean values, ZD958 had the lowest mean LA compared to the other two cultivars.

The TLNs of the cultivars selected for this study were widely variable. The degrees of variation of TLN in FK139, JNK728, and ZD958 were 1.5, 1.76, and 2.75 leaves, respectively. The TLN was significantly lower for the earlier and later sowing dates than when seeds were sown in the middle of the season.

**Figure 4:**
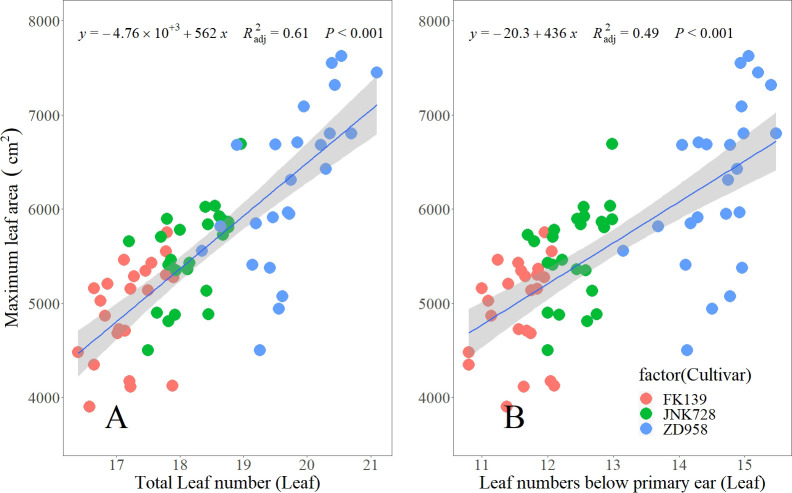
Relationship between leaf number variables and maximum leaf area at the silking stage. (**A, B** represent the relationship between the maximum leaf area and TLN, LB, respectively).

Across the three crop seasons, LB significantly differed among cultivars, and the changes in LB variation across sowing dates coincided with the changes in TLN variation. The variations in LB for FK139, JNK728, and ZD958 were 1.30, 1.28, and 2.33 leaves, respectively. The variation of TLNs and LBs could explain 61% and 49% of the maize maximum leaf area, respectively (**Figure 4**). Therefore, the difference of 1 or 2 leaves can significantly alter the maize canopy and this may affect biomass accumulation and grain yield.

LA also significantly differed between cultivars. The variations in LA for FK139, JNK728, and ZD958 were 0.88, 0.93, and 1.44 leaves, respectively. There were small differences in LA variation between the sowing, thus the LA remained relatively stable in comparison with the LB and TLN.

### The relationship between the three leaf counts

**Figure 5:**
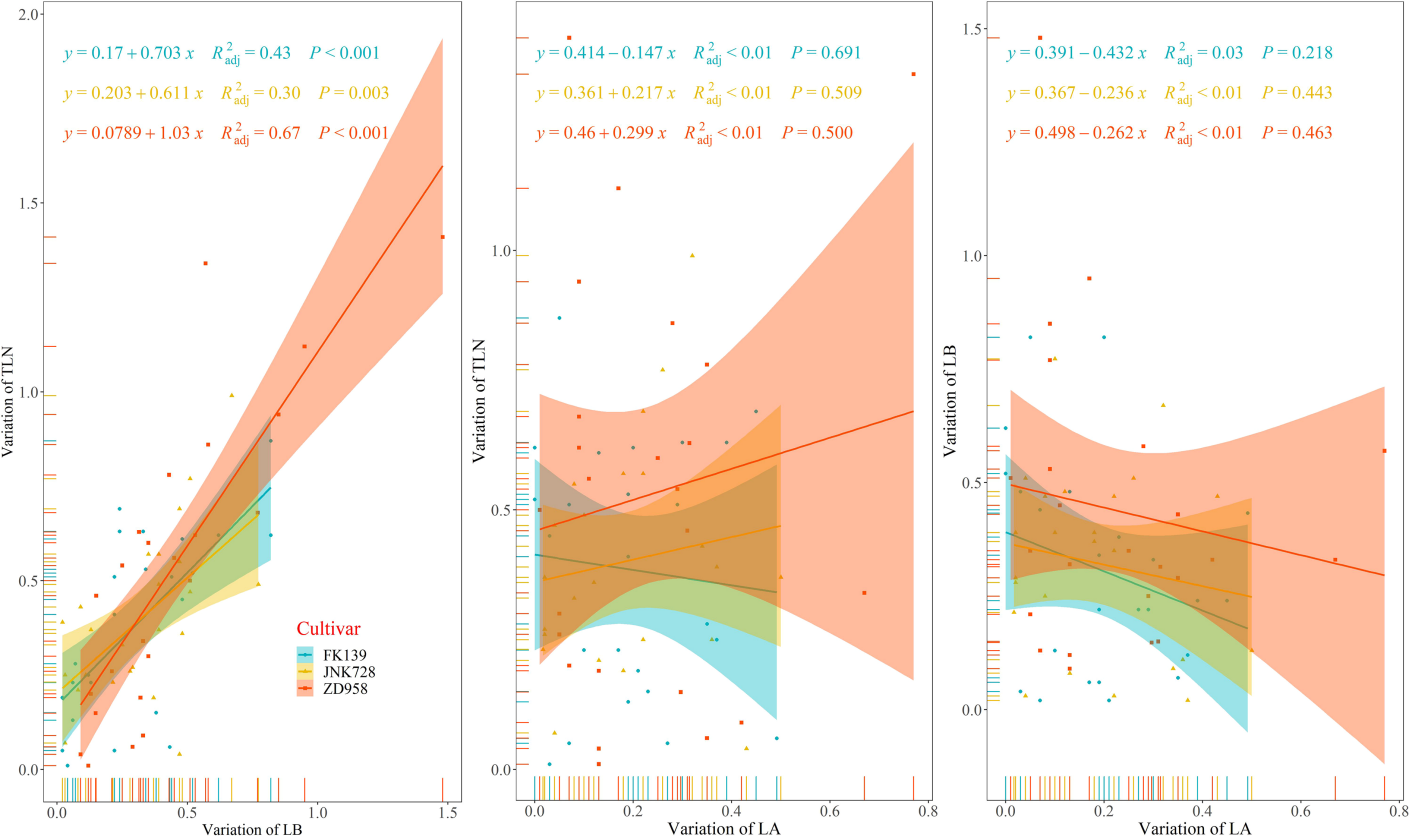
Relationships between variations in leaf number variables. Variation of LA is the absolute value of LA of a treatment minus the average value of LA of a cultivar; variation of LB is the absolute value of LB of a treatment minus the average value of LB of a cultivar; and variation of TLN is the absolute value of TLN of a treatment minus the average value of TLN of a cultivar. R^2^_adj_, adjusted R square.

As shown in **Figure 5**, the three cultivars exhibited wide variation in the three variables representing leaf number. Variation in TLN for FK139, JNK728, and ZD958 ranged from 0.01 to 0.87, 0.04 to 0.99, and 0.01 to 1.41 leaves, respectively. Variation in LB for FK139, JNK728, and ZD958 ranged between 0.04 to 0.82, 0.02 to 0.77, and 0.09 to 1.48 leaves, respectively. The respective ranges in variation of LA for FK139, JNK728, and ZD958 were 0 to 0.49, 0.02 to 0.50, and 0.01 to 0.77 leaves.

The correlation between variation in LB and variation in TLN was significant (*p<0.01*), and the adjusted R square values of FK139, JNK728, and ZD958 were 0.43, 0.30, and 0.67, respectively. In contrast, correlations between LA variation, TLN variation, and LB variation were insignificant at *p>0.05*. All of these results indicate that changes in TLN are more closely correlated with changes in LB.

### Effects of meteorological factors occurring during crucial growth stages on maize leaf number

**Figure 6:**
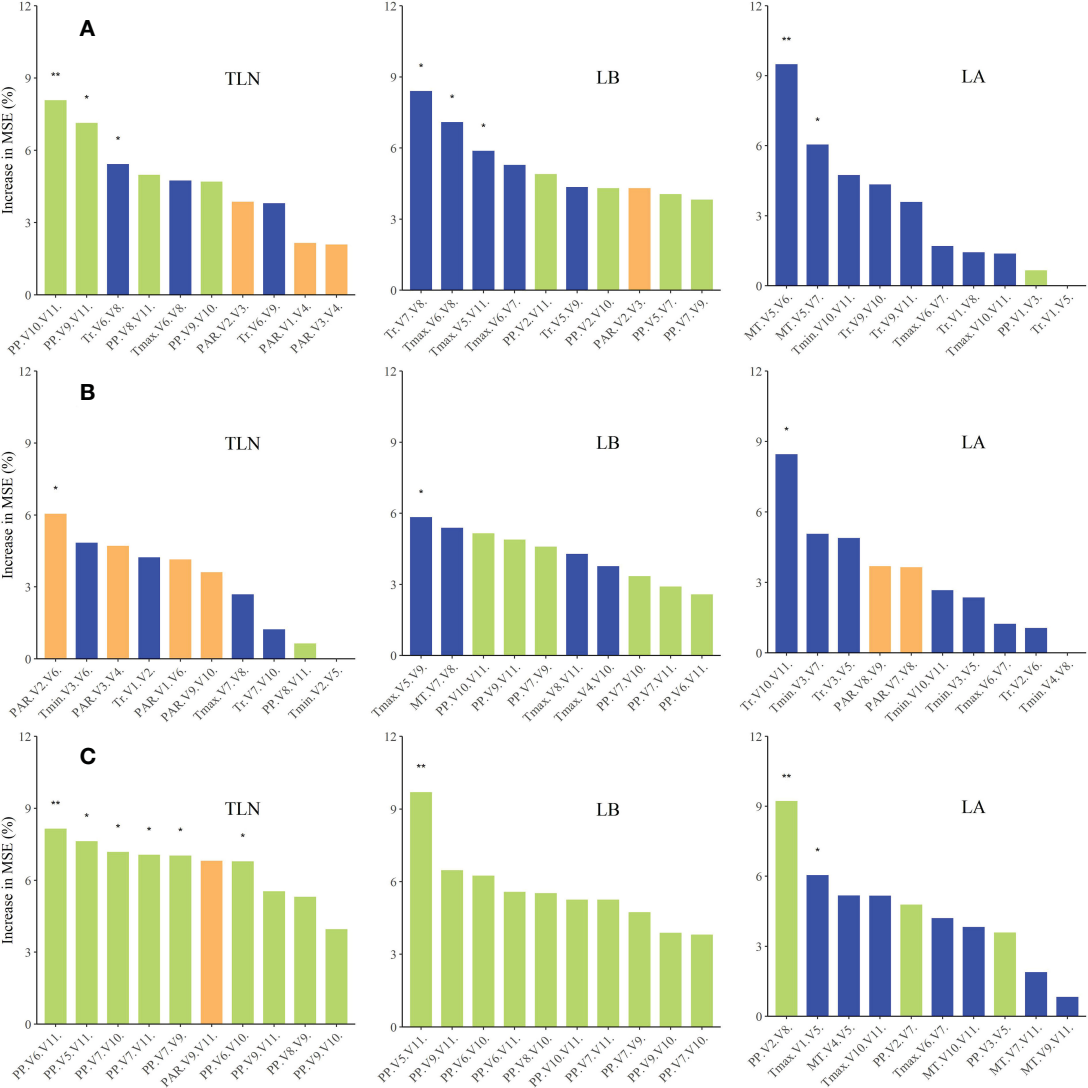
Relative contributions (percent increase of the MSE) of various meteorological factors as drivers of variations in TLN, LB, and LA of the three maize cultivars. Percent increases in the MSE of different variables were used to estimate the importance of these predictors, and higher MSE% values indicated greater importance of the predictors. MSE, mean squared error (A, FK139; B, JNK728; C, ZD958). * and ** are used to indicatesignificance levels at 0.01 < P < 0.05, 0.001 < P < 0.01, and P < 0.001,respectively. In addition, ns refers to P ≥ 0.05.

We used random forest regression analysis (**Figure 6**) to identify the major meteorological factors affecting changes in leaf number between the three cultivars and across different sowing dates. Variations in TLN, LB, and LA were jointly affected by photoperiod, PAR, and temperature-related factors. Variation of TLN was strongly associated with photoperiod and temperature-related factors. Variations in LB were primarily influenced by photoperiod, while variations in LA were mainly affected by temperature-related factors.

We also evaluated the responses of each cultivar to the various meteorological factors. Evidently, FK139 and JNK728 were more sensitive to photoperiod and temperature-related factors, while ZD958 was more sensitive to photoperiod during maize growth.

**Figure 7:**
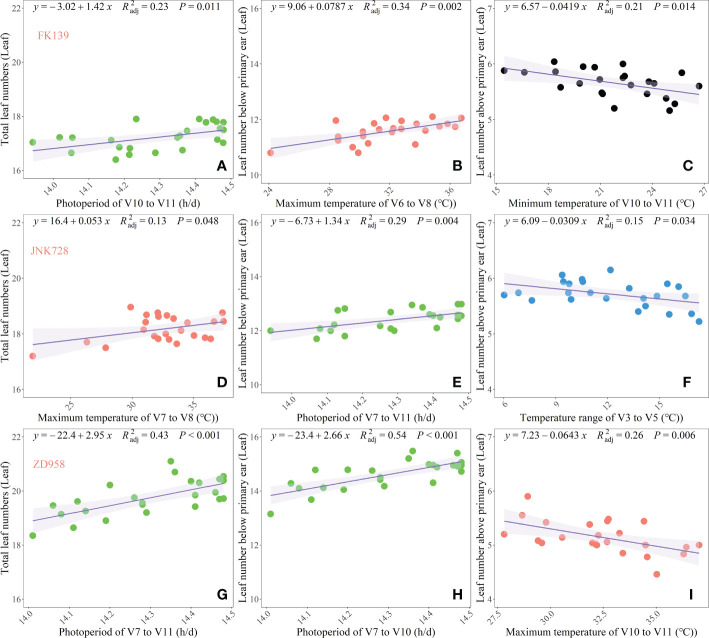
Key meteorological factors during crucial growth stages affecting leaf number responses of three maize cultivars. (**A–C**) respectively represent the TLN, LB, and LA of FK139; (**D–F**) respectively represent the TLN, LB, and LA of JNK728; and (**G–I**) respectively represent the TLN, LB, and LA of ZD958.

In addition, multivariate regression analysis was conducted to validate the observations of the random forest regression analysis and to clearly identify the most crucial meteorological factors that significantly affected the leaf number responses of the three cultivars (**Figure 7**). The TLNs of FK139, JNK728, and ZD958 were mainly affected by PP, Tmax, and PP in the V10 to V11 (R^2^_adj_=0.23, *P* < 0.05), V7 to V8 (R^2^_adj_ =0.13, *P* < 0.05) and V7 to V11 (R^2^_adj_ =0.44, *P* < 0.05) stages, respectively.

The LBs of FK139, JNK728, and ZD958 were mainly affected by the Tmax in the V6–V8 stages (R^2^_adj_ =0.34, *P* < 0.05), PP in the V7–V10 stages (R^2^_adj_ =0.29, *P* < 0.05) and PP in the V7–V10 stages (R^2^_adj_ =0.54, *P* < 0.05). The variation in TLN correlated in large part with the variation in LB, and photoperiod appeared to have the greatest influence on variations in TLN and LB. Photoperiod sensitivity, defined as the number of leaves that developed per hour increase of photoperiod, varied among cultivars, and sensitivities of TLN and LB to photoperiod ranged from 1.34 to 2.95 leaves h^-l^. Hence, photoperiod was the main driver of the variation observed in maize TLN.

Temperature-related factors during later growth stages primarily affected the LA of the three tested cultivars. These factors were the Tmin in the V10–V11 stage (R^2^_adj_ =0.21, *P* < 0.05), Tr in the V3–V5 stages (R^2^_adj_ =0.15, *P* < 0.05) and Tmax in the V10–V11 stage (R^2^_adj_ =0.15, *P* < 0.05). Overall, the variation of LA and the distribution of data for the three leaf number responses were mainly affected by temperature-related factors.

## Discussion

Consistent with the findings of previous research, we observed that adjusting the sowing date can expose a crop to a broad range of environmental conditions (Tsimba et al., 2013b; Bonelli et al., 2016; Zhang et al., 2019). As previously reported, maize TLN per plant varied relative to different sowing dates (Birch et al., 1998a; Tsimba et al., 2013b), and higher leaf numbers were recorded for the summer plantings (Chase and Nanda, 1967). The degrees of variation in TLN and LB were almost identical. Moreover, variations in TLN was primarily attributed to variations in LB. The degree of variation in LA was smaller than that of the LB or TLN, which has also been reported in earlier papers (Warrington and Kanemasu, 1983; Tollenaar, 1999; Liu et al., 2020). In contrast to the patterns we observed for variation in LA and LB in conventional maize, Subedi and Ma (2005) reported greater variation in LA than in LB for leafy hybrid maize.

According to the results of the random forest regression analysis, variations in TLN, LB, and LA of each cultivar were affected jointly by photoperiod, PAR, and temperature-related factors, largely confirming the conclusions of a previous study (Lejeune and Bernier, 1996), and photoperiod was a primary determinant of maize TLN and LB. Moreover, previous reports have shown that the effects of photoperiod on maize can occur as early as the V3 (leaf collar appearance) stage (Chen et al., 2015) and that maize leaf number is affected by photoperiod during the V5 and V7 stages (Tollenaar and Hunter, 1983). Excluding FK139, the results of this study showed that the photoperiod-sensitive interval for maize leaf number occurred approximately between stages V7 to V11, the duration of which was approximately 10.2 days; all durations were within the range found by a previous study where the photoperiod-induced phase varied from 0 to greater than 17 days (Rood and Major, 1980). One important finding of this research was that variations in LA were mainly affected by temperature-related factors, and to a lesser extent photoperiod and PAR, and the duration of the sensitive period for LA responses to temperature-related factors was estimated to be about 2.4 days. This finding is in line with findings of previous studies showing that temperature and photoperiod affected the variation of LA (Warrington and Kanemasu, 1983; Lejeune and Bernier, 1996), but is in contrast to previous results showing LA was not affected by photoperiod (Tollenaar, 1999).

Differentiation of maize leaves from stem apical meristems occurs before tassel initiation (Bonhomme et al., 1991), and the leaf number of maize plants is sensitive to photoperiod mainly between the time of emergence to tassel initiation. We assumed that maize was insensitive to photoperiod before the V7 stage (Wu et al., 2008; Craufurd and Wheeler, 2009). Environmental factors affect the leaf initiation rate and duration of leaf initiation, and these two leaf traits ultimately determine the final leaf number. The leaf initiation rate was constant from seedling emergence until tassel initiation (Warrington and Kanemasu, 1983). In this study, TLN and LB increased with increasing photoperiod, indicating that the photoperiod extension increased the leaf initiation duration and ultimately increased the maize leaf number (Birch et al., 1998a; Giauffret et al., 2000). On the other hand, variation of LA was mainly affected by temperature-related factors, suggesting that LA and LB are independently controlled at the genetic level (Li et al., 2016). The LAs of our three cultivars ranged from 4.46 to 6.15 leaves, which partly supports results of a previous report demonstrating that maize axillary meristems and leaf primordia at the apical meristem are initiated at the same rate but with a constant delay of 5.6–7.0 plastochrons (Lejeune and Bernier, 1996). Less variation in LA can reduce self-shading and shading by neighbors that inherently occurs in a maize crop (Subedi and Ma, 2005). Accordingly, a combination of environmental conditions affect the position of the maize primary ear and drive variations of LA and LB, consistent with findings that photoperiod, temperature, and irradiance may alter maize apical dominance which induces abortion of the topmost ear, affecting the primary ear position (Lejeune and Bernier, 1996; Lejeune et al., 1998).

Maize leaf number is an accessible morphological index with higher environmental plasticity, which can be used to determine maize population structure, and is also widely used as a measure of anthesis time, an indicator crucial to local environmental adaptation (Li et al., 2016; Zhang et al., 2019; Liu et al., 2020). Previous studies about the sensitive period during which maize leaf number is influenced by photoperiod and temperature-related factors are mainly based on developmental stages as defined by the number of visible leaf collars (Tollenaar and Hunter, 1983; Wu et al., 2008; dos Santos et al., 2022). In contrast, our results of sensitive periods are based on the number of visible leaf tips. The duration of successive appearances of new leaf tips was about 2 days that shorter than the period of successive appearances of new leaf collars, which is in accordance with findings involving twelve early maturing inbred corn lines reared under controlled-environmental conditions (Rood and Major, 1980). Differences in photoperiod during the sensitivity phase varied less than two hours but the maize leaf number varied by up to 5 leaves (Liu et al., 2013a; Liu et al., 2020). In our current study, a photoperiod during the sensitivity phase that varied less than one hour (0.7 h, **Figure 1**) led to significant variation in maize leaf, especially for the cultivar ZD958, the leaf number of which varied by up to 2.75 leaves, so the effects of photoperiod on leaf number may not be negligible. In addition, the sensitivities of TLN and LB to photoperiod (1.34 to 2.95 leaves h^-l^, **Figure 7**) were over the range reported by previous studies (Warrington and Kanemasu, 1983; Russell and Stuber, 1985; Bonhomme et al., 1991; Birch et al., 1998a), suggesting that basing the sensitive period on the number of visible leaf tips may more accurately reflect the real characteristics of some maize cultivars. The response in maize leaf number to photoperiod and temperature-related factors varied with season (Birch et al., 1998a), cultivar, and experimental location (Bonhomme et al., 1991). This study was conducted in the North China Plain, an area with a large sowing window (Huang et al., 2021) that differed in temperature, photoperiod, and radiative conditions. To the best of our knowledge this is the first study to use random forest regression analysis to determine major meteorological factors influencing the variability of maize leaf number and leaf positional distribution. A major advantage of the random forest regression is that its predictive power is not influenced by multicollinearity issues (Dormann et al., 2013). We also used a multiple regression model with variance partitioning analysis to validate the random forest analysis outcomes and identify key meteorological factors affecting variations in TLN, LB, and LA.

Results of this study have diverse implications for future research on maize physiology and eco-physiology, especially in modeling maize phenology and ontogeny (Bonhomme et al., 1991; Carberry and Abrecht, 1991; Birch et al., 1998a; Jones and Kirniry, 1986). Furthermore, adjusting the sowing date and selecting late-maturing cultivars, are two cost-effective options to contend with potential negative consequences of climate change (Liu et al., 2013b; Huang et al., 2020; Zhang et al., 2020). Adjusting the sowing date can expose maize to different environmental conditions; previous studies paid more attention to temperature, solar radiation, and water availability on maize production and ignored the effects of photoperiod, especially in relation to maize leaf number. It is true that some leaf numbers of some maize cultivars (FK139, JNK728) are insensitive to changing environment factors, while some cultivars such as ZD958 are sensitive to this change, which should be considered in studies and to develop models on the effects of adjusting the sowing date. Importantly, we determined that the photoperiod during the photoperiod-sensitive phase of maize development, which can be altered by adjusting sowing date, is one of the key factors affecting maize leaf number. A previous study indicated that the average photoperiod, ranging from just 13.7-15.6 h, during the photoperiod-sensitive phase of maize development was responsible for increasing total leaf numbers from 18.7 to 23.7 (Liu et al., 2013a). Therefore, more attention should be paid to variations of photoperiod during the photoperiod-sensitive phase of maize development. Moreover, based on our empirical evidence, when maize farmers select cultivars and adjust the sowing dates, they must consider each cultivar’s sensitivity to photoperiod to avoid dramatic decreases in leaf number. These reductions in leaf number may affect leaf area and the amount of incident radiation intercepted by the maize canopy, which ultimately influences maize assimilate production and maize grain yield (Lafitte and Edmeades, 1997; Liu et al., 2020). Further investigations into the effects of photoperiod on maize yield are necessary to improve the current understanding maize production and responses to different environmental conditions.

## Conclusions

Our study revealed that changing the sowing date can expose maize to different environmental regimes in terms of temperature, photoperiod, and solar radiation, and significantly influenced maize leaf numbers of the tested cultivars. The variation in TLN was attributed to variations in LB, both of which were greater than variations in LA. Variations in TLN and LB were mainly affected by photoperiod, particularly from growth stages V7 to V11, and this photoperiod sensitivity varied between cultivars. Variation in LA was mainly affected by temperature-related factors. Identifying key environmental conditions affecting leaf number variation in maize can provide theoretical references for assessing regional adaptations, developing breeding strategies, optimizing sowing dates of a given variety, and optimizing critical processes for maize crop modeling.

## Data availability statement

The raw data supporting the conclusions of this article will be made available by the authors, without undue reservation.

## Author contributions

HX: Data curation, formal analysis, resources, software, visualization, writing – original draft. BM: Data curation, formal analysis, resources, software, visualization, writing – original draft. KW: Writing – review & editing, project administration, methodology. JX: Data curation, visualization. PH: Data curation, visualization. SL: Conceptualization, funding acquisition, supervision, validation, writing – review & editing, project administration, methodology. RX: Conceptualization, funding acquisition, supervision, validation, writing – review & editing, project administration, methodology. All authors contributed to the article and approved the submitted version.
